# Role of image-guided fine-needle aspiration biopsy in the management of patients with splenic metastasis

**DOI:** 10.1186/1477-7819-5-13

**Published:** 2007-02-02

**Authors:** Luigi Cavanna, Antonio Lazzaro, Daniele Vallisa, Giuseppe Civardi, Fabrizio Artioli

**Affiliations:** 1 Medical Oncology and Hematology Department, Hospital of Piacenza, 29100 Piacenza, Italy; 2 Internal Medicine Division, Hospital of Fiorenzuola, 29017 Fiorenzuola (Piacenza), Italy; 3 Medical Oncology Unit, Hospital of Carpi, 41012 Carpi (Modena), Italy

## Abstract

**Background:**

Splenic metastases are very rare and are mostly diagnosed at the terminal phase of the disease or at the time of autopsy. The cytohistological diagnosis, when done, is made prevalently by splenectomy. Reports on splenic percutaneous biopsies in the diagnosis of splenic metastasis are fragmentary and very poor.

The aims of this study are to analyse retrospectively the accuracy, safety and the clinical impact of ultrasound (US)-guided fine-needle aspiration biopsy (UG-FNAB) in patients with suspected splenic metastasis.

**Methods:**

A retrospective analysis of 1800 percutaneous abdominal biopsies performed at our institute during the period from 1993 to 2003 was done and 160 patients that underwent splenic biopsy were found. Among these 160 patients, 12 cases with the final diagnosis of solitary splenic metastases were encountered and they form the basis of this report. The biopsies were performed under US guidance using a 22-gauge Chiba needle. All the patients underwent laboratory tests, CT examination of the abdomen and chest, US examination of abdomen and pelvis.

**Results:**

There were 5 women and 7 men, median age 65 years (range 48–80). Eight patients had a known primary cancer at the time of the diagnosis of splenic metastasis: 3 had breast adenocarcinoma, 2 colon adenocarcinoma, 2 melanoma and 1 lung adenocarcinoma. Four patients were undiagnosed at the time of the appearance of splenic metastasis and subsequent investigations showed adenocarcinoma of the lung in 2 patients and colon adenocarcinoma in the remaining 2. There was a complete correspondence between the US and Computed Tomography (CT) in detecting focal lesions of the spleen.

The splenic biopsies allowed a cytological diagnosis of splenic metastasis in all the 12 patients and changed clinical management in all cases.

Reviewing the 160 patients that underwent UG-FNAB of the spleen we found no complications related to the biopsies.

**Conclusion:**

These results indicate that UG-FNAB is a successful technique for diagnosis of splenic metastasis allowing an adequate treatment of the affected patients.

## Background

Clinically evidence of splenic metastasis is very rare and the metastasis of the spleen is mostly diagnosed at the terminal phase of the disease or at the time of autopsy. However in last decades with the development of imaging techniques such as ultrasound (US) and computerized tomography (CT) the diagnostic approaches of abdominal organs have changed, since US and CT can easily detect focal lesions of these organs.

The detection of focal defects in the spleen at US and CT examination in patients with malignancy may suggest the presence of metastasis, however US, CT, as well as MR imaging and radionuclide scans cannot differentiate metastasis from other splenic lesions such as infections or lymphoma and a cytological diagnosis can be required since it may effect therapeutic decisions and prognosis [1,2]. With the development and refinement of new guidance modalities for percutaneous biopsies, many investigator have reported favourable results with biopsies of various abdominal organs [3-5], however there have been only a few reports detailing percutaneous biopsy of the spleen [6-8]. Most reports on percutaneous biopsy of the spleen concern malignant lymphomas or abscesses [9-12], while to our knowledge, reports on solitary splenic metastasis diagnosed with guided-percutaneous biopsies are fragmentary or very poor [6,13-16].

The aims of this study were to evaluate the accuracy, safety and the role of splenic biopsy in the management of patients with suspected solitary splenic metastasis.

## Patients and methods

A retrospective analysis of 1,800 abdominal percutaneous guided biopsies performed at Medical Oncology and Hematology Department, Hospital of Piacenza between 1993 and 2003 was done and 160 patients that underwent splenic biopsy were found. Among these 160 cases, 12 patients with the final diagnosis of solitary splenic metastasis were encountered and form the basis of this report. There were not 12 consecutive patients, but selected cases ultimately proven to be positive for splenic metastasis. Solitary splenic metastasis was defined as focal single lesion in the spleen parenchyma.

All the patients underwent laboratory tests, CT examination of the abdomen and chest, US examination of abdomen and pelvis. The diameter of the spleen were measured with US, the splenic focal lesions were measured and recorded. Splenomegaly was defined by clinical examination, when the spleen was palpable and by US and CT examination when the spleen measured over 13 cm in long axis.

The indication criteria used to perform splenic biopsies in this set of patients were: a) to perform a definite diagnosis for undiagnosed patients; b) to establish with certainty the only site of metastasis; c) to ascertain the progression in patients with metastatic but stable disease.

The splenic biopsies in the 160 patients were performed as previously reported [9,10,12], briefly ultrasonically guided fine-needle aspiration biopsy (UG-FNAB) was done choosing the most convenient route, avoiding the pleura and vessels. A 22-gauge Chiba needle was introduced into the focal lesion of the spleen under US guidance using a real-time scanner with a puncturing probe; if the material obtained was not considered adequate for diagnosis after a rapid cytologic evaluation by the cytopathologist present during biopsy as previously reported [17], the procedure was immediately repeated. Material aspirated was smeared on slides; Papanicolau and May Grunwald-Giemsa staining methods were routinely used on the cytologic smears. The diagnostic evaluation of the specimen was made by an experienced cytologist, and the confirmation of the diagnoses was based on histologic sampling, surgery, clinical, US and CT follow-up of longer than six months.

Conditions necessary to do the biopsy were: informed consent, prothrombin activity more than 50%, platelet count higher than 70.000/μl and 12 hours of fasting before the biopsy. The procedure was done in some instance on an outpatient basis (2 hours in bed with ice applied and compressed on the abdominal wall). Twenty-four hours after the splenic biopsy, US abdominal examination and a complete blood count were undertaken to ascertain whether complications had occurred. We check also for late complications with a contact to patients. Premedication was not given, any complications were recorded and analgesia given as appropriate.

## Results

Disease status, patients characteristics at the time of biopsy and results of splenic biopsies of the twelve patients with splenic metastases are listed in table 1.

**Table 1:** Patients characteristics and results of splenic fine-needle aspiration biopsy

**Patients number**	**Sex/Age**	**Primary cancer**	**Disease status at the time of biopsy**	**Presentation**	**US pattern, Lesion's size, splenic size**	**Other cancer localization**	**Results of splenic biopsy**	**Treatment**	**Response**
1	F/58	Breast ADC	Stable disease	Asymptomatic US detection	Hypoechoic 3.5 cm normal	Liver	metastasis	CT	PR
2	F/65	Breast ADC	Stable disease	Asymptomatic US detection	Hypoechoic 1.5 cm normal	Bone	Metastasis	CT and RT	PR
3	F/70	Breast ADC	Solitary splenic recurrence	Asymptomatic US detection	Hypoechoic 2.5 cm normal	None	Metastasis	Splenectomy and CT	CR
4	M/65	Colon ADC	Solitary splenic recurrence	Asymptomatic US detection	Echogenic 4 cm normal	None	Metastasis	Splenectomy and CT	CR
5	F/72	Colon ADC	Solitary splenic recurrence	Asymptomatic US detection	Hypoechoic 3.5 cm normal	None	Metastasis	CT and RT	CR
6	M/80	Colon ADC	Solitary splenic presentation	Asymptomatic US detection	Hypoechoic 3 cm normal	None	Metastasis	Splenectomy and surgical treatment of colon cancer	CR
7	M/48	Colon ADC	Solitary splenic presentation	Abdominal pain, weight loss	Hypoechoic 4 cm normal	None	Metastasis	Splenectomy and surgical treatment of colon cancer	CR
8	M65	Lung ADC	Splenic recurrence	Asymptomatic US detection	Hypoechoic 2 cm normal	None	Metastasis	CT	PR
9	M/57	Lung ADC	Solitary splenic presentation	Asymptomatic US detection	Hypoechoic 2.5 cm normal	None	Metastasis	Splenectomy and surgical treatment of lung cancer	CR
10	M/76	Lung ADC	Solitary splenic presentation	Asymptomatic US detection	Echogenic with hypoechoic halo 6 cm normal	Mediastinal lymphonodes	Metastasis	CT and RT	PR
11	M/44	Melanoma	Stable disease	Asymptomatic US detection	Hypoechoic 4 cm normal	Lung and lymphonodes	metastasis	RT	PD
12	F/56	melanoma	Stable disease	Asymptomatic US detection	Hypoechoic 8 cm normal	liver	metastasis	none	PD

ADC: adenocarcinoma; CT: chemotherapy; RT: radiotherapy; PR: partial response; CR: complete response; PD; progressive disease

There were 5 women and 7 men, median age 65 years (range 48–80). Eight patients had a know primary cancer at the time of diagnosis of splenic lesion: three had breast adenocarcinoma, 2 colon adenocarcinoma, 2 melanoma, 1 non small cell lung cancer (adenocarcinoma); the splenic metastasis was metachronous in all these 8 patients. Four patients were undiagnosed at the time of the appearance of splenic lesion. Subsequent investigations showed adenocarcinoma of the lung in 2 patients (patient number 9 and 10), and colon adenocarcinoma in two (patient number 6 and 7). Among the 8 patients with a known primary cancer the splenic lesion was the only site of metastasis in 4 patients (patients number 3, 4, 5, 8), while in the remaining 4 patients it represented the sign of progression since these patients, 2 with breast cancer (patients number 1, 2) and 2 with melanoma (patients number 11, 12), had metastatic but stable disease at the time of US evidence of splenic lesion. In the 4 patients previously undiagnosed the splenic lesion was the isolated site of metastasis in 3 (patients number 6, 7, 9), while patient number 10 had mediastinal lymphonodes involvement. The clinical presentation of splenic metastasis was asymptomatic in 11 patients (91.6%) and disclosed by radiologic technique during clinical investigations or follow-up; 1 patient presented abdominal discomfort, weight loss and fever (patient number 7).

The US pattern of the splenic lesions was hypoechoic in the majority of the patients (10/12, 83.3%), ecogenic and ecogenic with hypoechoic halo in the remaining two patients (16.7%).

The focal lesions of the spleen measured in diameter from 1.5 to 8 cm, the spleen showed normal size in all the patients.

The total number of patients examined in this study period with a bioptic verified cancer disease was 1620. All these patients were evaluated with CT/US and splenic metastases were identified only in 12 cases, so the incidence of splenic metastases in this cohort of patients was 0.74%.

The splenic biopsies allowed a cytological diagnosis in all the 12 patients with splenic metastasis and these results were sufficient to define a specific therapeutic approach in all the patients. Five patients underwent splenectomy; in addition three of them were successfully treated with surgery for their primary malignancy: colorectal cancer (patient number 6 and 7) and non-small cell lung cancer (patient number 9). Two patients were also treated with chemotherapy (patients number 3 and 4). The remaining 7 patients received a non surgical treatment: two chemotherapy (patients number 1 and 8), three chemotherapy and radiation (patients number 2, 5 and 10), one radiotherapy alone (patient number 11) and one patient received no further treatment since the splenic metastasis was asymptomatic and the patient showed progressive disease after a second line of chemotherapy (patient number 12).

The responses to the treatment were complete remission in 6 patients (50%), partial response in 4 (33.3%) and progression in 2 (16.7%).

The other 148 patients (pts) that underwent splenic biopsy by UG-FNAB showed the following disease: malignant disease 122 patients (non-Hodgkin's lymphoma 112 pts, Hodgkin's disease 10 pts), non malignant disease 26 patients (abscess 11 pts, granulomatosis 5 pts, tuberculosis 6 pts, atypical cyst 4 pts).

To calculate the sensitivity and positive predictive value of FNAB, the numbers of true-positive (TP), true-negative (TN), false-positive (FP) and false-negative (FN) results were recorded (table 2). The malignant FNABs were considered as true positives (TP) in cases where subsequent evaluations revealed a malignancy, and they were considered false positives (FP) when no malignancy was found. The benign FNAB was considered as a true negative (TN) if they were confirmed as benign lesions and false negative (FN) in cases of proven malignancy. From these numbers, the following statistical values have been calculated: sensitivity in percentage as [(TP/TP + FN) * 100], positive predictive value (PPV) in percentage as [(TP/TP + FP)* 100] and overall accuracy as [(TP + TN)/(TP + FP + FN + TN) * 100]. Applying this statistical analysis in all the 160 cases we found that splenic biopsy showed a sensitivity of 98.4% and a positive predictive value of 99.2%. No complications were recorded.

**Table 2:** Overall results of 160 splenic fine-needle aspiration biopsy.

**Results**	**N°**	**Statistical analysis**	**%**
True positive (TP)	126	Sensitivity	98.4
True negative (TN)	29		
False negative (FN)	2	Positive predictive value (PPV)	99.2
Insufficient	2		
False positive (FP)	1	Overall accuracy	98.1
total	160		

## Discussion

In patients with non-lymphomatous malignancy splenic metastases are infrequent with an autopsy evidence ranging from 2 to 8% [18,19]. In these patients however evidence of widespread metastatic disease is usually present, whereas isolated splenic metastases are rare [20]. For this reasons the diagnostic problem of splenic metastasis has traditionally been of low clinical impact in oncology patients. It must be emphasized, as reported by Keogan *et al *[2], that with the increased use of immunosuppressive agents, focal lesions in some patients may be related to infection rather than malignancy. As a result, an increase in demand for splenic fine-needle aspiration has been reported from oncology centers [6]. Recently Mohammadi and Calne described a case with splenic metastasis and reviewed the cases with solitary splenic metastases reported in the literature and approximately 50 cases were found [1].

Approximately 60% of these reported cases appear to be associated with gynaecologic cancers; in this series the highest association with solitary splenic metastases is represented by ovarian and endometrial tumors. Colorectal cancers also represent an high association (11%) of the primary tumor sites. Furthermore, the majority of the primary tumors are of histologic type of adenocarcinoma.

In our series the primary cancers are represented by breast, 3 cases (25%), colon 4 cases (33.3%), lung 3 cases (25%), melanoma 2 cases (16.6%), and according with the cases previously reported the most frequent histologic type is adenocarcinoma: 10 cases (83.3%). Our patients with solitary splenic metastasis reported here differ in some aspects with the cases previously reviewed [1]: none of our patients had a palpable splenomegaly compared with 11/53 (20.8%); in 11 of our 12 patients (91.7%) the splenic involvement was asymptomatic compared with 40/53 (75.5%) [1].

All our patients were pathologically diagnosed by ultrasonically guided fine needle aspiration biopsy, while only two patients reported in the review of literature were diagnosed by fine-needle aspiration [1]. In our series splenectomy was performed only in five patients, and the remaining seven cases were all, but one (patient number 12 with disease progression), treated with chemotherapy and/or radiotherapy. Splenectomy was initially planned in all the 7 patients with isolated splenic metastases, however two of them refused splenectomy (patients number 5 and 8).

Our results show that splenic metastasis can be successfully diagnosed by fine-needle aspiration biopsy. It must be emphasized that in the 160 patients of our institution that underwent splenic biopsy, this technique showed a sensitivity of 98.4% and a positive predictive value of 99.2% without complications. These findings support the suggestion that this technique is valuable for diagnosis and may be underused [2,6], since there is a paucity of North American and European reports concerning FNAB for splenic focal lesion and above all for metastatic disease involving the spleen.

Silverman *et al *[13] reported a series of 11 FNAB of the spleen in patients with carcinoma (4 patients) or haematological malignancies (7 patients). FNAB confirmed metastatic carcinoma in 3 patients, malignancy in additional 3 patients, spleen infections in 3 patients (candida, aspergillus and abscess) and extensive necrosis in the remaining patient. Only one hemorrhagic complication was noted following splenic biopsies.

More recently Caraway and Fanning [6] reported the results of FNAB performed on 50 patients of whom 40 had a previous diagnosis of malignancy (23 lymphoproliferative disorders, 13 carcinomas, 3 melanomas and 1 sarcoma). The cytologic diagnoses included 22 cases positive for malignancy (10 lymphomas, 9 metastatic carcinomas, 2 metastatic melanomas, and 1 sarcoma). No major complications were associated with the FNAB procedure, one patient developed a pneumothorax that resolved spontaneously.

We previously reported the results of splenic fine-needle tissue-core biopsy on 46 patients with lymphoma and we concluded that the technique was safe and useful for the diagnosis, staging, and follow-up of malignant lymphomas [10].

More recently we reported the results of a multicenter Italian study concerning 398 splenic biopsies: on overall accuracy rate of 98.1% and a low complication rate (no death cases, less than 1% for major complications, and 5.2% for all complications) were recorded [12]. In the present series of 160 patients that underwent UG-FNAB of the spleen, the procedure had a treatment clinical impact in 155 (96.9%). In the remaining 5 cases there was a delay in treatment defining because 3 patients showed false results (1 false positive and 2 false negative in low-grade NHL) and 2 cases with Hodgkin disease obtained inadequate results.

## Conclusion

Our findings showing an overall accuracy of 98.1% calculated on 160 patients, without complications, are favourable compared with the remaining literature [2,6,8,12,13]. It must be emphasized that the correct diagnosis of the focal splenic lesions is becoming an important issue in both undiagnosed and diagnosed patients, due to same emerging factors such as the improvement in to the ability of diagnosing imaging techniques to detect parenchymal focal lesions and prolonging of life expectancy in patients with malignant lymphomas and some solid tumor. Therefore, the indications for splenic biopsy are expanding and our results reported here show that UG-FNAB of the spleen is an effective, safe and cheap technique for a definite pathological diagnosis.

## Competing interests

The author(s) declare that they have no competing interests.

## Authors' contributions

**LC**, carried out the bioptical procedures and contributed to the design of the study; revised and finally approved the manuscript for been published.

**AL**, carried out the bioptical procedures and contributed to the design of the study; gathered the data, drafted the manuscript and critically revised it;

**DV **carried out the bioptical procedures and contributed to the design of the study; gathered the data, drafted the manuscript and critically revised it;

**GC **carried out the bioptical procedures and contributed to the design of the study;

**FA **revised and finally approved the manuscript for been published.

All authors read and approved the manuscript.
